# Estimated pulse wave velocity (ePWV) as a potential gatekeeper for MRI-assessed PWV: a linear and deep neural network based approach in 2254 participants of the Netherlands Epidemiology of Obesity study

**DOI:** 10.1007/s10554-021-02359-0

**Published:** 2021-07-25

**Authors:** Max J. van Hout, Ilona A. Dekkers, Ling Lin, Jos J. Westenberg, Martin J. Schalij, J. Wouter Jukema, Ralph L. Widya, Sebastiaan C. Boone, Renée de Mutsert, Frits R. Rosendaal, Arthur J. Scholte, Hildo J. Lamb

**Affiliations:** 1 Department of Cardiology, Leiden University Medical Center, Albinusdreef 2, 2333 ZA Leiden, The Netherlands; 2 Department of Radiology, Leiden University Medical Center, Albinusdreef 2, 2333 ZA Leiden, The Netherlands; 3 Department of Epidemiology, Leiden University Medical Center, Albinusdreef 2, 2333 ZA Leiden, The Netherlands

**Keywords:** Magnetic resonance imaging, Pulse wave velocity, Prediction modelling

## Abstract

Pulse wave velocity (PWV) assessed by magnetic resonance imaging (MRI) is a prognostic marker for cardiovascular events. Prediction modelling could enable indirect PWV assessment based on clinical and anthropometric data. The aim was to calculate estimated-PWV (ePWV) based on clinical and anthropometric measures using linear ridge regression as well as a Deep Neural Network (DNN) and to determine the cut-off which provides optimal discriminative performance between lower and higher PWV values. In total 2254 participants from the Netherlands Epidemiology of Obesity study were included (age 45–65 years, 51% male). Both a basic and expanded prediction model were developed. PWV was estimated using linear ridge regression and DNN. External validation was performed in 114 participants (age 30–70 years, 54% female). Performance was compared between models and estimation accuracy was evaluated by ROC-curves. A cut-off for optimal discriminative performance was determined using Youden’s index. The basic ridge regression model provided an adjusted R^2^ of 0.33 and bias of < 0.001, the expanded model did not add predictive performance. Basic and expanded DNN models showed similar model performance. Optimal discriminative performance was found for PWV < 6.7 m/s. In external validation expanded ridge regression provided the best performance of the four models (adjusted R^2^: 0.29). All models showed good discriminative performance for PWV < 6.7 m/s (AUC range 0.81–0.89). ePWV showed good discriminative performance with regard to differentiating individuals with lower PWV values (< 6.7 m/s) from those with higher values, and could function as gatekeeper in selecting patients who benefit from further MRI-based PWV assessment.

**Supplementary Information:**

The online version contains supplementary material available at 10.1007/s10554-021-02359-0.

## Introduction

Cardiovascular disease (CVD) is still the leading cause of death in the world, despite the efforts and expenses that have been put in improving cardiovascular care [1, 2]. Therefore, increasing the accuracy of cardiovascular risk assessment is imperative to guide the efficacy and efficiency of treatment. Pulse wave velocity (PWV), a measure of aortic stiffness, is a risk factor for cardiovascular morbidity and mortality and has been added to the most recent ESC hypertension guideline for cardiovascular risk assessment [3–5]. Carotid-femoral PWV (cfPWV) using applanation tonometry and magnetic resonance imaging (MRI) are commonly used techniques for PWV assessment. cfPWV is easy to use, however less accurate due to the inability to accurately assess aortic length and thereby resulting in a systematic overestimation of PWV as compared to MRI-PWV [6]. Additionally, cfPWV is unable to assess local aortic PWV and measurements in obese patients can be challenging. In contrast, MRI provides the most accurate non-invasive assessment of PWV [6]. However, MRI also has several disadvantages, as it is not widely available, requires local technical expertise, is relatively expensive and time consuming, which limits the application of MRI-based PWV in clinical care. An adequate estimation of PWV (ePWV) using an equation based on clinical determinants that are easily assessable could reduce the amount of MRI scans needed. As such, ePWV would be widely available for cardiovascular risk assessment, which could contribute to the implementation of PWV in clinical care.

Traditionally, prediction models were developed using linear regression, however potential non-linear associations between cardiovascular risk factors and PWV could hamper the predictive performance of linear regression models. Deep neural networks (DNN) use a dense network of layers containing multiple neurons that can operate linearly as well as non-linearly. DNN simulates a biological neural network and in theory should provide improved model performance as compared to linear regression in complicated medical prediction modelling, which has been illustrated by previous neural network estimation equations that outperformed traditional linear regression [7, 8]. To our knowledge, a DNN-based prediction model for MRI-based PWV has not yet been reported in literature. Several determinants such as age, sex, smoking, obesity, diabetes, hypertension and dyslipidaemia are known to influence PWV and would be relevant determinants for both linear and DNN models to estimate PWV [9, 10]. High PWV values are likely more difficult to accurately predict, as the interplay of the different risk factors becomes more complex. We postulate that lower PWV values can be accurately predicted by both linear and DNN based models, but that higher PWV values may still require MRI for accurate assessment. Determining a cut-off that can discriminate reliably between lower and higher values could provide a threshold at which point MRI assessment is needed. Our objective was therefore to develop both a linear and DNN-based equation to estimate MRI-based PWV (ePWV), and to determine the cut-off which provides optimal discriminative performance between lower and higher PWV values.

## Methods

### Development and internal validation sample

The present study is a cross-sectional analysis of the baseline measurements in the Netherlands Epidemiology of Obesity (NEO) study (see https://www.lumc.nl/org/neo-studie/ for more information); a population-based, prospective cohort study in 6671 individuals aged 45–65 years [11]. Men and women living in the greater area of Leiden (the Netherlands) were invited to participate in the study if they were aged between 45 and 65 years and had a self-reported body mass index (BMI) of ≥ 27 kg/m^2^. In addition, all inhabitants from one municipality (Leiderdorp) were invited to participate irrespective of their BMI, allowing for a reference distribution of BMI (n = 1671). Participants completed general questionnaires on demographic, lifestyle and clinical information. At the baseline visit, all participants underwent an extensive physical examination including anthropometry, blood pressure measurements and blood samples. Approximately 35% of the participants were randomly selected for abdominal MRI including PWV (except those with potential contraindications for MRI). We aimed to develop a prediction model that applies to a population without known CVD, as this population will benefit the most from accurate cardiovascular risk assessment. Therefore, participants with overt CVD (myocardial infarction, angina, congestive heart failure, stroke, or peripheral vascular disease) were excluded. The Medical Ethical Committee of the Leiden University Medical Center (LUMC) approved the design of the study and all participants gave their written informed consent.

### External validation sample

The participants of the  MAGNA VICTORIA study were used for the external validation, the study population and design have been previously described [12]. This is a prospective double blind clinical trial aimed at evaluating effects of liraglutide on cardiovascular end-points assessed using cardiac MRI, including PWV. For the external validation we used the baseline measurements including anthropometric measures, blood pressure and PWV assessed using MRI.

### Magnetic resonance imaging

In the development cohort, MRI was performed on a 1.5 Tesla scanner (Philips Medical Systems, Best, the Netherlands) [11, 13]. In the external validation cohort, participants were scanned on a 3 Tesla scanner (Ingenia, Philips, Best, The Netherlands) [12]. Retrospective ECG-gated gradient-echo sequence with velocity encoding was performed during free breathing to assess aortic flow. Imaging parameters of development sample: field-of-view 300 mm, rectangular field-of-view percentage 90%, echo time 2.8 ms, repetition time 4.8 ms, flip angle 20°, acquired voxel size 2.34 × 2.34 × 8.00 mm, velocity encoding 200 cm/s. Imaging parameters of external validation sample: field-of-view 350 mm, rectangular field-of-view percentage 80%, echo time 2.5 ms, repetition time 4.4 ms, flip angle 20°, acquired voxel size 2.8 × 2.8 × 8.00 mm, velocity encoding 200 cm/s. Maximum velocity–time curves provided the arrival time of the systolic pressure wave. The foot of the systolic wave front was detected automatically using in-house developed software, by assessing the intersection point of the horizontal diastolic flow and the upslope of the systolic wave front, modelled by a linear regression along the upslope from the flow values between 20 to 80% of the range. Through-plane flow measurements were performed at the level of the pulmonary trunk perpendicular to the ascending aorta and just above the bifurcation of the abdominal aorta. For pathlength assessment, a gradient-echo multi-slice (8 slices) oblique-sagittal scout image was acquired to capture the entire aorta (field-of-view 225 × 225 × 40 mm, echo time 1.85 ms, repetition time 3.70 ms, flip angle 55°, acquired voxel size 1.8 × 1.8 × 5.0 mm). The aortic path length between the measurement sites was measured using MASS software (Medis, Leiden, The Netherlands). Aortic path length divided by transit time between arrival of the systolic wave front at these sites was used to calculate PWV in m/s.

### Statistical analysis

**Fig. 1:**
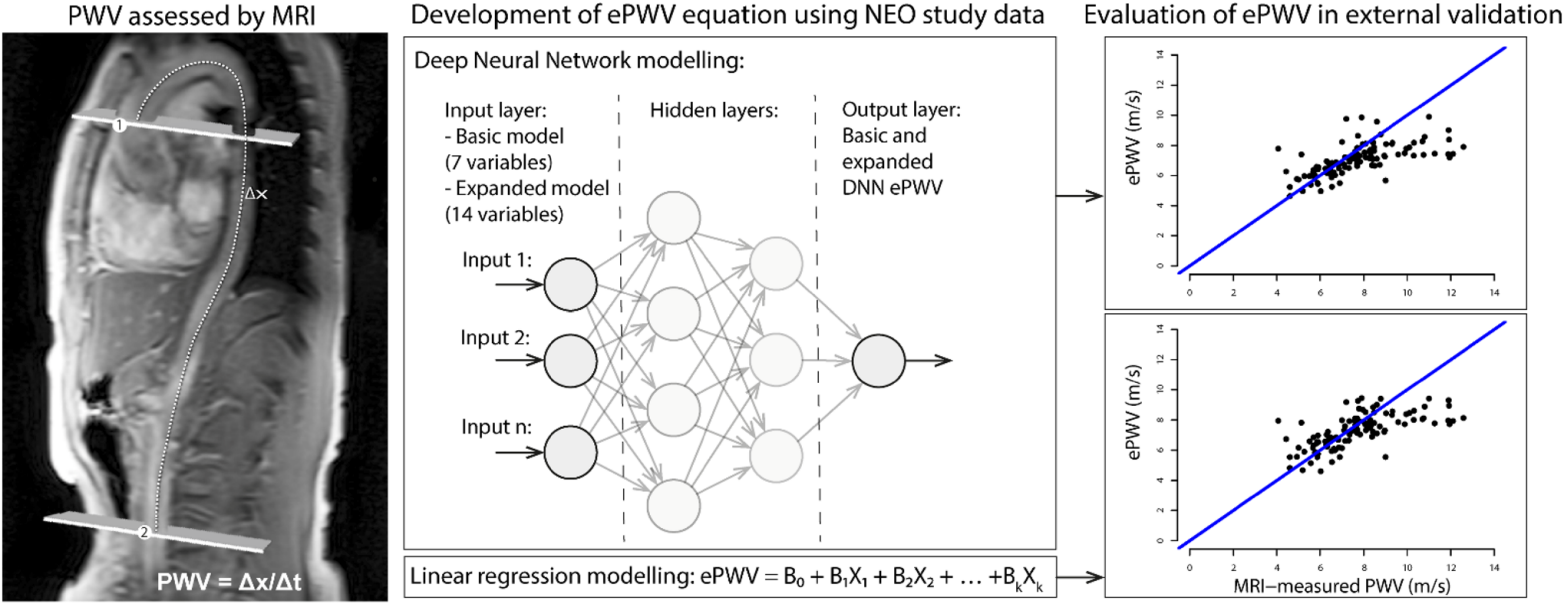
Study overview: left panel: PWV measurement by MRI; middle panel: illustration of prediction model development using deep neural networks and linear ridge regression; right panel: illustration of ePWV model performance evaluation in the external validation dataset

We performed a complete case analysis on all participants who had available PWV measurements. To optimise the potential applicability of our model we developed 2 models, a basic model with few predictors which is easier to use in clinical practice and a more extensive model which is possibly more accurate. The pre-specified variables were selected based on literature, clinical relevance and anticipated availability in most clinical settings. We developed a basic model to estimate PWV based on few predictors (age, sex, height, weight, heart rate, systolic and diastolic blood pressure), and an expanded model requiring HbA1c, total cholesterol, use of antihypertensive (beta blockers, alpha blockers, calcium-channel blockers, ACE-inhibitors/ AT2-antagonist, vasodilators or diuretics), antidiabetic or cholesterol lowering medication and smoking status including pack years in addition to the seven basic parameters. Both linear regression and DNN models were used to develop the basic and expanded equations (Fig. 1). Model performances were assessed using the adjusted R^2^, bias, mean absolute error (MAE), root mean squared error (RMSE) and Bland–Altman plots.

### Linear model

For the linear regression models a multivariable ridge regression was performed. The average tuning parameter that minimized the mean squared error (MSE) in 10 repeats of tenfold cross-validation was used. Internal validation was performed using bootstrapping with 150 repetitions, in which all modelling steps were repeated, from which optimism-corrected performance parameters were calculated [14].

#### Deep Neural Network

The Keras package for R was used for DNN model development. First, 90% of the male and female participants were randomly selected to form the training sample, the remaining 10% formed the internal validation sample. Second, data pre-processing was performed where the 7 predictors of the basic and 14 predictors of the expanded model formed the model input. MRI-PWV was coded as the training target. A sequential model with several hidden layers was used to develop a neural network. Additional layers with a dropout function were added to the model to test whether this would result in improved internal validation performance through a reduction of overfitting [15]. The input layers consisted of the 7 and 14 predictors of the basic and expanded model. The single output layer provided the estimated PWV. Each hidden layer used an activation function that could be activated in different ways, which function to use was determined in the training process. The mean squared error (MSE) was used as the loss function for the learning algorithm. Model training and tuning was based on the shape of the learning curve, adjusted R2, RMSE and MAE. Tuning of the validation split, epochs and batch size, activation functions, amount of hidden layers and neurons, were performed to obtain the optimal adjusted R2 and minimal RMSE and MAE.

#### Cut-off

We determined the cut-off that provided optimal discriminative performance between lower and higher PWV values in the development data. To determine the optimal cut-off we used Youden’s Index, which is based on specificity and sensitivity of the prediction model to differentiate between values above and below a specific cut-off [16]. The dataset was dichotomised into values above and below cut-offs across the PWV range, the cut-off that provided the highest Youden Index was used.

#### External validation

The equations derived by ridge regression and DNN were applied in the external validation sample. Receiver-operating characteristics (ROC) curves were computed for measured-PWV categorized into low and high. The area under the ROC (AUC), specificity, sensitivity and accuracy (percentage of agreement between ePWV and measured-PWV) were calculated to test how often ePWV falls in the same category as the measured-PWV. The AUC’s were compared using DeLong’s test. Differences in means between models were tested using a paired t-test. We predefined an AUC of > 0.7 as acceptable and > 0.8 as excellent [17]. Development and analysis of ePWV models were performed using R version 3.6.1.

## Results

**Fig. 2:**
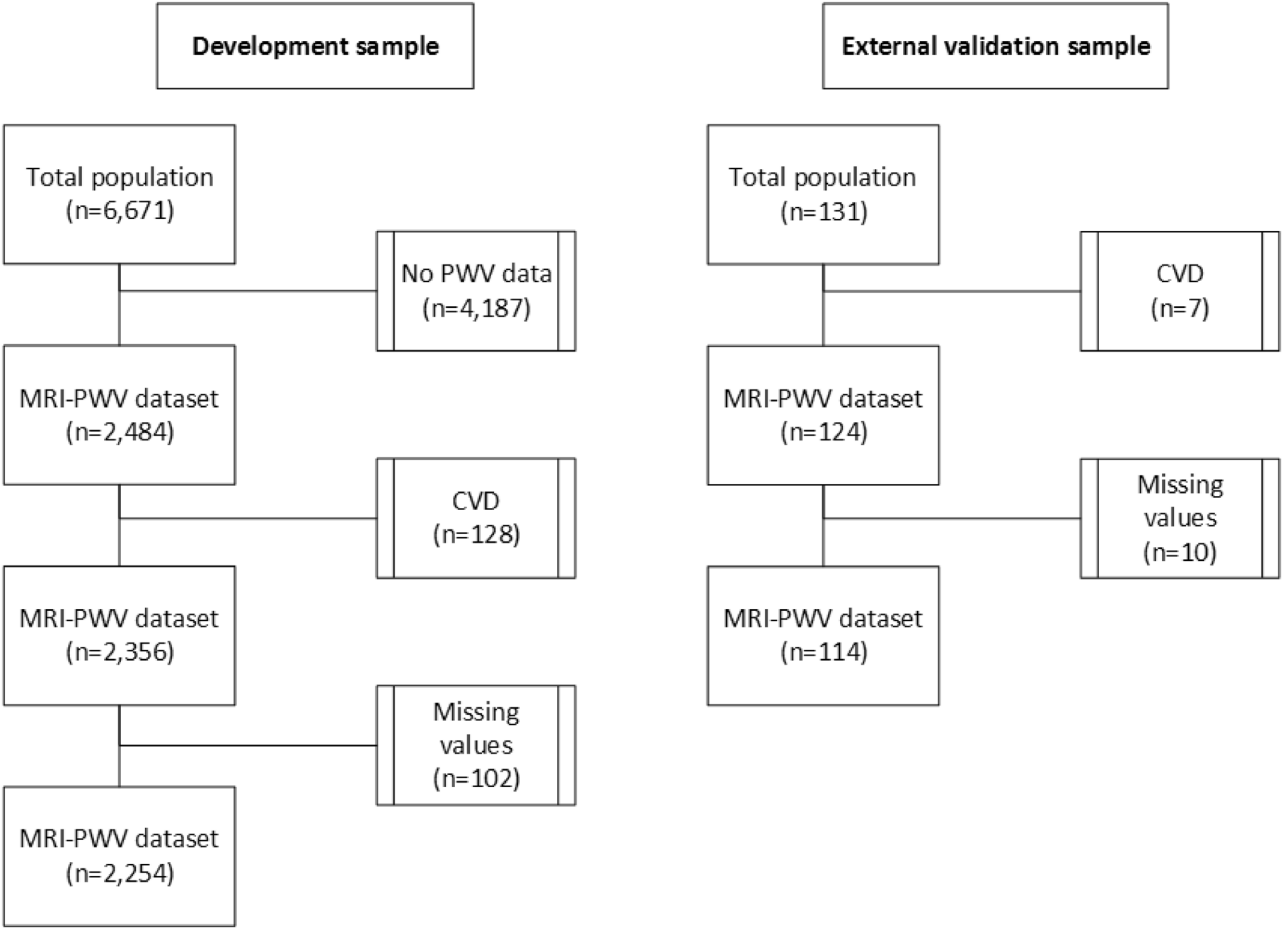
Flow chart for sample selection. *CVD* cardiovascular disease, *PWV* pulse wave velocity

Characteristics of the development sample and external validation sample are shown in Table 1. From the 2484 participants of the development sample who had available MRI-PWV data, 2254 participants free from CVD without missing data were selected (Fig. 2, age 45–65 years, 51% male, mean PWV 6.63 ± 1.27 m/s). From the 131 participants of the external validation sample, 114 participants free from CVD without missing data were selected (age 30–70 years, 54% female, mean PWV 7.71 ± 1.88 m/s).

**Table 1:** Characteristics of the study populations

	Development/internal validation sample		External validation sample	
	Men n = 1170	Women n = 1084	Men n = 50	Women n = 64
Characteristics				
Age (years)	55.8 ± 6.1	55.5 ± 5.8	56.8 ± 9.0	53.3 ± 9.1
Length (m)	1.81 ± 0.07	1.66 ± 0.06	1.77 ± 0.07	1.63 ± 0.07
Weight (kg)	95.8 ± 12.7	81.5 ± 14.3	86.9 ± 15.8	73.3 ± 14.6
BMI (kg/m^2^)	29.3 ± 3.4	29.4 ± 4.9	27.6 ± 4.2	27.7 ± 5.5
BSA (m^2^)	2.19 ± 0.17	1.93 ± 0.19	2.06 ± 0.21	1.81 ± 0.19
Total body fat (%)	28.4 ± 5.6	41.3 ± 6.0	26.0 ± 5.2	39.4 ± 7.4
Systolic blood pressure (mmHg)	136.7 ± 15.3	128.4 ± 17.3	138.3 ± 14.8	132.9 ± 21.8
Diastolic blood pressure (mmHg)	86.3 ± 10.1	83.7 ± 10.3	87.8 ± 9.6	80.5 ± 9.9
Heart rate (beats/min)	67.7 ± 10.9	70.7 ± 10.6	69.7 ± 11.3	72.8 ± 13.0
Pulse wave velocity (m/s)	6.6 ± 1.2	6.7 ± 1.3	8.2 ± 1.8	7.4 ± 1.9
Smoking (%)				
Never	413 (35.3)	432 (39.9)	27 (54.0)	44 68.8)
Former	558 (47.7)	533 (49.2)	5 (10.0)	8 (12.5)
Current	199 (17.0)	119 (11.0)	18 (36.0)	12 (18.8)
Pack years	11.3 ± 16.0	8.6 ± 13.3	5.0 ± 10.6	3.9 ± 9.3
Glucose lowering medication (%)				
No	1113 (95.1)	1052 (97.0)	21 (42.0)	30 (46.9)
Oral medication	44 (3.8)	27 (2.5)	13 (26.0)	9 (14.1)
Insulin	4 (0.3)	1 (0.1)	0 (0)	1 (0)
Oral medication and insulin	9 (0.8)	4 (0.4)	16 (32.0)	25 (39.1)
Lipid lowering medication (%)	169 (14.4)	91 (8.4)	25 (50.0)	22 (34.4)
Medication for hypertension (%)	301 (25.7)	277 (25.6)	21 (42.0)	22 (34.4)
Total cholesterol (mmol/L)	5.64 ± 1.05	5.85 ± 1.09	5.09 ± 1.23	5.02 ± 1.05
Triglycerides (mmol/L)	1.63 ± 1.03	1.27 ± 0.74	1.59 ± 1.47	1.46 ± 0.94
HDL (mmol/L)	1.26 ± 0.32	1.62 ± 0.42	1.37 ± 0.37	1.57 ± 0.48
LDL (mmol/L)	3.63 ± 0.96	3.65 ± 1.01	2.95 ± 1.12	2.78 ± 0.90
Glucose (mmol/L)	5.78 ± 1.16	5.53 ± 0.89	6.84 ± 2.19	6.52 ± 2.22
HbA1c (%)	5.45 ± 0.60	5.40 ± 0.42	7.07 ± 1.59	6.85 ± 1.55
Creatinine (umol/L)	86.1 ± 14.2	69.5 ± 10.6	84.1 ± 15.6	61.2 ± 9.2

Data are shown as n (%) or mean ± SD

*BMI* body mass index, *BSA* body surface area, *HDL* high-density lipoprotein, *LDL* low-density lipoprotein

## DNN model training

The finished basic DNN model was constructed by three hidden layers containing 8, 8 and 4 neurons respectively. The final expanded DNN model was constructed by 2 hidden layers containing 15 and 8 neurons respectively. Addition of a dropout function with various dropout rates was tested, but did not result in improved model performance in internal validation and was therefore not used. The Rectified Linear Unit (ReLu) activation function was used for all layers in both models. The used learning rate was “optimizer_rmsprop (lr = 0.001)”. Training of the model revealed that epochs = 100, batch size = 16, and validation split = 0.1, resulted in the optimal model.

## DNN and Ridge regression model development and internal validation

Development and internal validation model performance parameters are discussed in the supplemental material and can be found in supplemental Table 1. Estimation of PWV using the basic and expanded DNN model can be performed at https://epwv.shinyapps.io/webpage/. The generated equations of the Ridge regression models are presented in Table 2.

**Table 2:** Regression equations for the ePWV

Regression model	Equation
Basic	$$\begin{gathered} \left( {0.{\text{7}}0{\text{4}}*{\text{height}}} \right)\; + \;\left( {0.{\text{282}}*{\text{sex}}} \right)\; + \;\left( {0.0{\text{88}}*{\text{age}}} \right)\; + \;\left( {0.0{\text{17}}*{\text{heart rate}}} \right) \hfill \\ + \;\left( {0.0{\text{15}}*{\text{systolic BP}}} \right)\; + \;\left( {0.0{\text{15}}*{\text{diastolic BP}}} \right)\;{-}\;\left( {0.00{\text{2}}*{\text{weight}}} \right)\;{-}\;{\text{3}}.{\text{853}} \hfill \\ \end{gathered}$$
Expanded	$$\begin{gathered} \left( {0.{\text{669}}*{\text{height}}} \right)\; + \;\left( {0.{\text{275}}*{\text{sex}}} \right)\; + \;\left( {0.{\text{146}}*{\text{glucose}}\;{\text{lowering medication}}} \right) \hfill \\ + \,\left( {0.0{\text{85}}*{\text{age}}} \right)\, + \,\left( {0.0{\text{72}}*{\text{HbA1c}}} \right)\,{-}\,\left( {0.0{\text{68}}*{\text{medication for hypertension}}} \right) \hfill \\ + \,\left( {0.0{\text{52}}*{\text{lipid lowering medication}}} \right)\,{-}\,\left( {0.0{\text{31}}*{\text{smoking}}} \right) \hfill \\ + \,\left( {0.0{\text{16}}*{\text{diastolic BP}}} \right)\, + \,\left( {0.0{\text{16}}*{\text{heart rate}}} \right)\, + \,\left( {0.0{\text{14}}*{\text{systolic BP}}} \right) \hfill \\ {-}\,\left( {0.00{\text{3}}*{\text{weight}}} \right)\, + \,\left( {0.00{\text{1}}*{\text{total cholesterol}}} \right)\, + \,\left( {0.00{\text{1}}*{\text{pack years}}} \right)\, - {\text{ 3}}.{\text{896}} \hfill \\ \end{gathered}$$

*BP* blood pressure

Based on the Youden index, the optimal cut-off to discriminate between low versus high PWV was found to be 6.7 m/s for both linear and DNN models.

## Model performance in the external validation sample

**Fig. 3:**
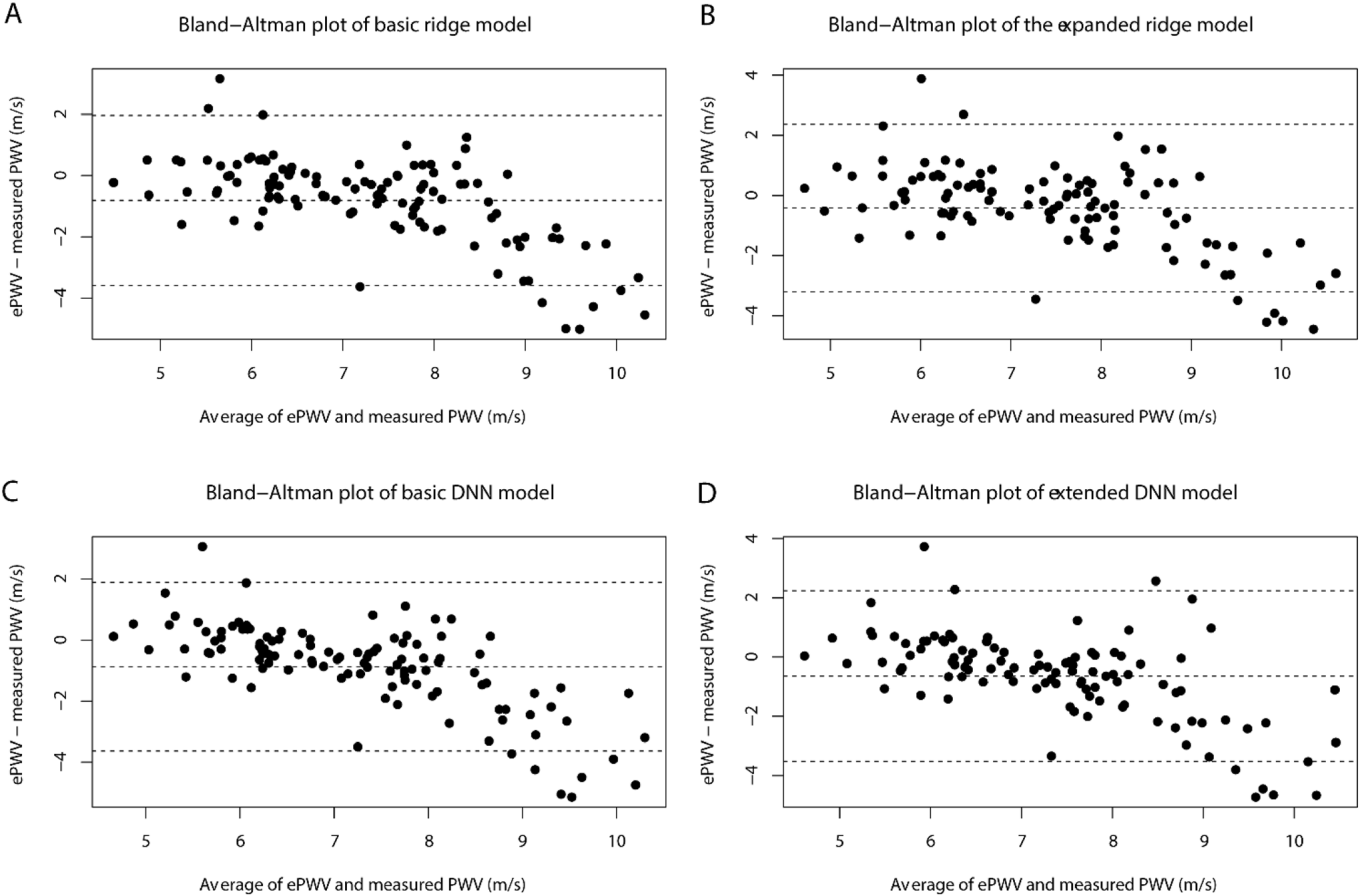
Bland–Altman plots of ePWV versus measured-PWV. **A** Basic ePWV ridge regression model. **B** Expanded ePWV ridge regression model. **C** Basic ePWV DNN model. **D** Expanded ePWV DNN model

**Fig. 4:**
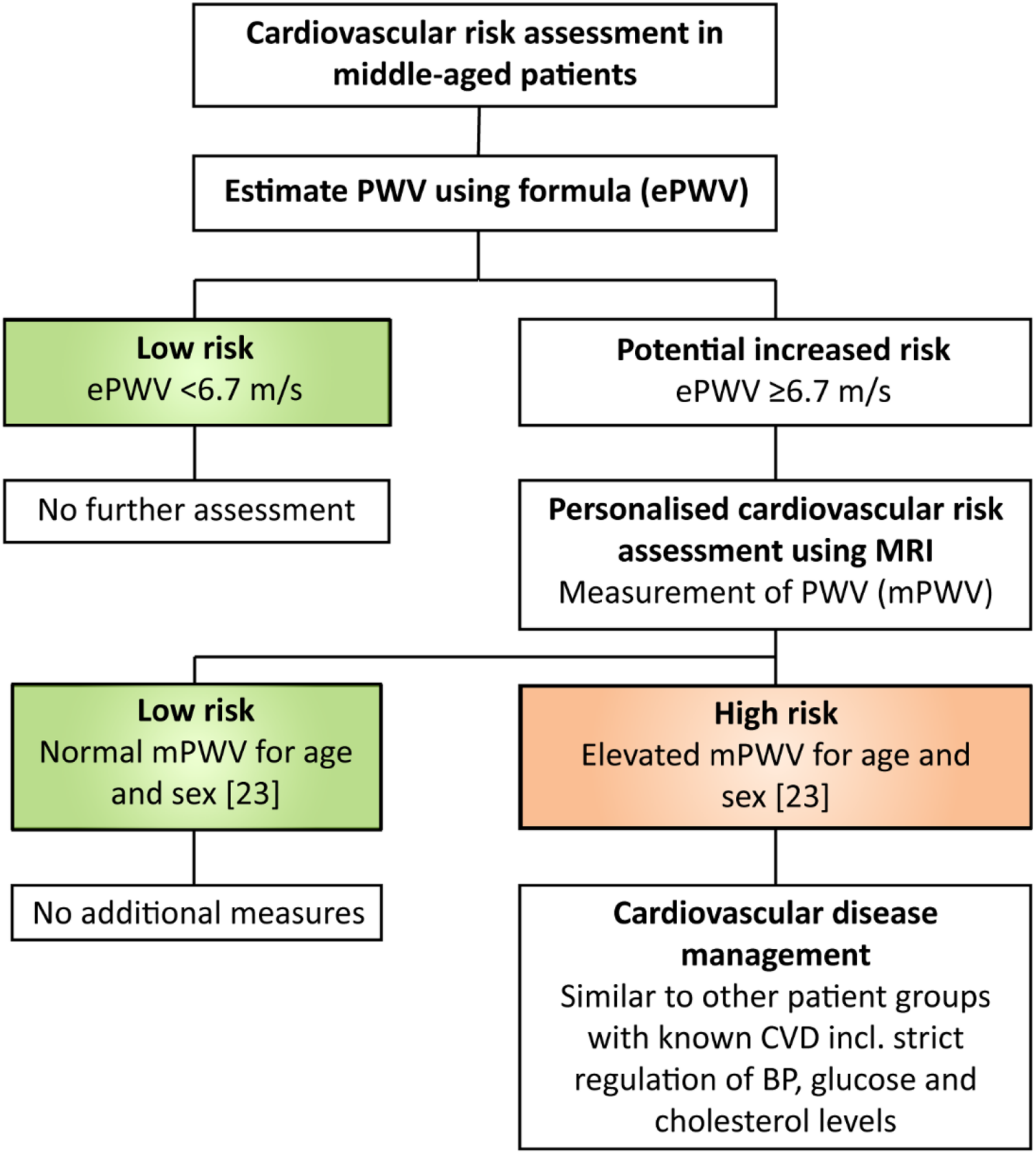
A suggestion for cardiovascular risk management using ePWV and MRI-PWV. *BP* blood pressure, *CVD* cardiovascular disease, *ePWV* estimated pulse wave velocity, *mPWV* measured pulse wave velocity

Performance of the four models in the external validation dataset are shown in Table 3. The expanded ridge regression model provided the best performance measures in the external validation sample with an adjusted R^2^ of 0.29. As is illustrated in the Bland–Altman plots in Fig. 3, lower PWV values showed good agreement, whereas predictions of higher PWV values were less accurate with a systematic underestimation of the measured-PWV. The AUC, sensitivity, specificity and accuracy of the four models estimating PWV < 6.7 m/s, ≥ 6.7 m/s are presented in Table 4. Because of limited sample size of the external validation sample, this was not used to calculate prediction parameters. Discriminating ability of ePWV for values < 6.7 versus ≥ 6.7 m/s had AUC values ranging from 0.81–0.89 with high accuracy (0.84–0.88) for both basic and expanded models in ridge as well as DNN-based models. The AUC of the expanded ridge regression model was lower than the basic model (p = 0.03). No other differences were found in the comparison of ridge and DNN models. A suggestion for cardiovascular risk management using ePWV and MRI-PWV is provided in Fig. 4.

**Table 3:** External validation of the regression models and DNN models

	Adjusted R^2^	RMSE (m/s)	MAE (m/s)	Bias (m/s)
Linear ridge regression based models				
Basic model	0.20	1.62	1.14	0.80
Expanded model	0.29	1.47	1.07	0.42
DNN based models				
Basic model	0.17	1.65	1.17	0.87
Expanded model	0.22	1.60	1.13	0.64

*DNN* deep neural network, *MAE* mean absolute error, *RMSE* root mean sum of squared errors

**Table 4:** External validation receiver operating characteristic analysis

			AUC (95% CI)	Sens. (95% CI)	Spec. (95% CI)	Accuracy (95% CI)
PWV < 6.7 m/s	Ridge	Basic	0.89 (0.84–0.95)	0.95 (0.82–0.99)	0.84 (0.74–0.92)	0.88 (0.80–0.93)
Versus ≥ 6.7 m/s		Expanded	0.81 (0.73–0.90)	0.73 (0.56–0.86)	0.90 (0.80–0.96)	0.84 (0.76–0.90)
	DNN	Basic	0.87 (0.80–0.93)	0.92 (0.78–0.98)	0.81 (0.70–0.90)	0.85 (0.77–0.91)
		Expanded	0.87 (0.80–0.94)	0.86 (0.71–0.95)	0.87 (0.77–0.94)	0.87 (0.79–0.93)

*AUC* Area under the receiver operating characteristic (ROC) curve, *DNN* deep neural network, *PWV* pulse wave velocity

### Discussion

In this analysis of 2254 participants of the  NEO study, we developed ridge regression and DNN based prediction models to estimate MRI-assessed PWV using cardiovascular risk factors and anthropometric measures. External validation was performed in 114 participants of the  MAGNA VICTORIA study. All ePWV models provided good discriminative performance with regard to differentiating individuals with lower PWV (< 6.7 m/s) from those with higher PWV values. ePWV combined with measured MRI-PWV could reduce the amount of MRI scans needed, while increasing the availability of accurate cardiovascular risk assessment. To the best of our knowledge, this is the first reported MRI-based ePWV model.

### Basic and expanded ePWV models

Prediction modelling in vascular medicine allows for early, accessible and affordable estimation of cardiovascular risk beyond traditional risk factors. A previous ePWV model has been developed based solely on blood pressure and age to predict the cfPWV [10]. This model showed similar predictive performance as compared to our model including limited prediction of high PWV values (R^2^ range 0.27–0.45) [10, 18]. However, a post-hoc analysis of the SPRINT study showed that estimated cfPWV predicts outcome beyond the Framingham Risk Score and found better survival in participants whose estimated cfPWV responded to antihypertensive treatment independent of systolic blood pressure [19]. Albeit these findings may suggest a role for markers of aortic stiffness as effective treatment targets in patients with hypertension, the systemic underestimation of high PWV values highlights that ePWV should be used with caution in clinical practice and could be particularly useful as a gatekeeper for additional testing.

Our basic and expanded models that estimate MRI-based PWV showed similar performance as the previous model developed to estimate cfPWV [10]. As was also shown in the previous study, accurate estimation was particularly difficult for the high measured-PWV range. Higher PWV values may be more difficult to predict due to the more complex interplay of different risk factors, as is illustrated by the increased variability of PWV with age irrespective of blood pressure [20]. Nonetheless, high predictive performance should not be the only focus in assessment of clinical prediction models. A good clinical example is the estimated glomerular filtration rate (eGFR), which has taken up a central role in estimating kidney function despite the suboptimal predictive performance of eGFR models [21]. Regardless of its limitations, eGFR is accurate enough to discriminate between different stages of renal dysfunction, which is most important in a clinical setting. In cases where more accuracy is aspired, measurement of GFR using gold standard invasive techniques is recommended [22]. The developed prediction models in this study all showed good discriminative performance between individuals with lower (< 6.7 m/s) versus higher (≥ 6.7 m/s) PWV values. Albeit normal values of PWV are age dependent, in the middle-aged general population PWV values below 6.7 m/s correspond to the low end of the distribution for both men and women [23]. This indicates a possible gatekeeper function for ePWV when applied in the middle-aged population, where an ePWV < 6.7 m/s has a high likelihood of low aortic stiffness and warrants no additional vascular stiffness assessment. In such a scenario, ePWV values ≥ 6.7 m/s would indicate the need for additional measurement of PWV by MRI for accurate assessment of vascular morbidity and the associated cardiovascular risk, whereas for ePWV values < 6.7 m/s no additional measurement of PWV would be needed. In current guidelines the role of MRI-based PWV is unclear, even though it provides the most accurate non-invasive assessment of aortic stiffness [5]. ePWV as a pre-selection tool could aid in the clinical implementation of MRI-based PWV as this reduces the number of scans, considering MRI is only needed for accurate measurement of higher PWV values. A suggestion for cardiovascular risk management using ePWV and MRI-PWV is provided in **Fig. ****4****.** As such, a combination of ePWV with MRI-assessed PWV might be a safe and cost effective strategy for more widely available accurate cardiovascular risk assessment, however this remains area for future research. In the future it would also be interesting to investigate the prospective validation of 10 year CVD outcome in the NEO population [11].

In our models we used easily identifiable and broadly available markers associated with PWV, of which age and blood pressure provide the most weight in the regression function as is consistent with previous literature [24]. Arterial stiffness is known to develop differently over life in men and women, however in middle-aged populations previous studies often found no important sex differences, as is also observed in our model [23, 25]. The variation of PWV with heart rate has been documented extensively and subsequently heart rate provided substantial weight in the regression equation [26]. It is somewhat remarkable that body weight did not provide a greater impact as compared to height given the known association between obesity and PWV, although there have been studies that show the greater importance of height in PWV assessment [27]. Our basic model performed reasonable in the development data, however in external validation the expanded model performed better indicating a beneficial effect of the additional parameters on generalizability. Besides generalizability, addition of laboratory results and cardiovascular risk factors associated with PWV did not improve model performance.

#### Ridge regression versus deep neural network

Ridge regression and DNN models showed relatively similar performance parameters in the training data, however in the internal and external validation performance parameters of the ridge regression models were slightly better. This is likely due to overfitting, which is a known issue in DNN models [28, 29]. To account for overfitting in DNN, a dropout function was added; however, this did not result in improved model performance [15]. Neural networks have been used in medical analysis with varying success, as evidenced in numerous studies in different areas of medicine [7, 30, 31]. A common explanation for the added predictive performance of neural networks is the amount on non-linearity present between the used variables, however the consistency of machine learning models has recently been challenged [32].

#### Limitations

There are several limitations that need consideration. For MRI-based measures, it can be difficult to obtain large sample sizes, although new large size population studies and improved automated image analysis are providing new opportunities. The sample size of 2254 that was used in this study, was relatively limited compared to what is commonly used in DNN applications, however smaller sample sized studies have found added value of neural networks. This limitation was particularly illustrated in our external validation, an important analysis to test generalizability which often is not performed [33]. Due to the intrinsic limitation of DNN to overfit the data, we added a dropout function which did not result in improved performance in internal validation. Moreover, we restricted model variables to those widely available and the model was developed in a largely white population aged 45–65, limiting generalizability in other age groups and ethnicities.

## Conclusion

The current study is the first to report a prediction model to estimate MRI-based PWV. ePWV showed good discriminative performance with regard to differentiating individuals with lower PWV values (< 6.7 m/s) from those with higher values, and could function as gatekeeper in selecting patients who benefit from further MRI-based PWV assessment. Thereby, MRI scan time and healthcare costs might be saved.

## Supplementary Information

Below is the link to the electronic supplementary material.

Supplementary file1 (DOCX 265 kb)

## Data Availability

Due to the privacy of the participants of the NEO study and legal reasons, we cannot publicly deposit the data. Also, NEO study participants did not sign consent to make their data publicly available. Data will be made available upon request to interested qualified researchers. Data requests should be sent to the NEO Executive Board which can be contacted via https://www.lumc.nl/org/neostudie/contact/.
