# Supplementary material for: Estimated pulse wave velocity (ePWV) as a potential gatekeeper for MRI-assessed PWV: a linear and deep neural network based approach in 2254 participants of the Netherlands Epidemiology of Obesity study
Source: Int J Cardiovasc Imaging. 2021 Jul 25;38(1):183–93. doi: 10.1007/s10554-021-02359-0 (PMC8818644; doi:10.1007/s10554-021-02359-0)

*Ridge regression model*

Performance of the basic and expanded ridge regression and DNN models are shown in **table S1**. The basic ridge regression model, consisting of 7 predictors (age, sex, height, weight, pulse, systolic and diastolic blood pressure), showed an adjusted correlation with an R^2^ of 0.33 as is shown in **table S1**. The expanded ridge regression model, which included HbA1c, total cholesterol, use of antihypertensive, antidiabetic or cholesterol lowering medication and smoking status including pack years in addition to the seven basic parameters, did not show any added predictive performance. Mean measured-PWV and ePWV of the basic and expanded ridge regression models were all similar (6.63 m/s). As is illustrated in the Bland-Altman plots of the basic and expanded model in **figure S1**, the higher measured-PWV predictions were less accurate, with a systematic underestimation of the PWV.

*Ridge regression model performance in the internal validation bootstrap sample*

The Bootstrap sample produced similar performance parameters as the original basic and expanded models (**table S1**).

*DNN model*

Results of the DNN models are shown in **table S1**. The basic model provided an adjusted R^2^ of 0.33, similar to the ridge regression model, however with higher bias of 0.11 m/s. The expanded model provided a slightly higher adjusted R^2^ of 0.34 with lower bias of 0.06 m/s. Mean basic ePWV (6.74 ± 0.64 m/s) was higher than the expanded ePWV (6.70 ± 0.66 m/s; p<0.001) and both were higher compared to the measured-PWV (p<0.001; p=0.005 respectively). Similar as in the ridge regression, the Bland-Altman plots of the basic and expanded models illustrate the limited predictive performance for higher measured-PWV values (**figure** **S1**). Estimation of PWV using the basic and expanded DNN model can be performed at https://epwv.shinyapps.io/webpage/.

*DNN model performance in the internal validation sample*

The split sample internal validation showed inferior performance parameters as compared to the original basic and expanded DNN models (**table S1**). The basic model provided an adjusted R^2^ of 0.22 with a bias of 0.12 m/s. The expanded model had an adjusted R^2^ of 0.18 with a bias of 0.07 m/s.

**Table S1** Performance of the regression models and DNN models

| **Linear ridge regression based models** | | | | | |
| --- | --- | --- | --- | --- | --- |
|  |  | Adjusted R^2^ | RMSE (m/s) | MAE (m/s) | Bias (m/s) |
|  | **Basic model** | 0.33 | 1.03 | 0.74 | <0.001 |
|  | Bootstrap validation | 0.33 | 1.04 | 0.74 | 0.002 |
|  | **Expanded model** | 0.33 | 1.03 | 0.74 | <0.001 |
|  | Bootstrap validation | 0.33 | 1.04 | 0.75 | 0.002 |
| **DNN based models** | | | | | |
|  |  | Adjusted R^2^ | RMSE (m/s) | MAE (m/s) | Bias (m/s) |
| 8.8.4 | **Basic model** | 0.33 | 1.02 | 0.77 | 0.11 |
|  | Split sample validation | 0.22 | 1.27 | 0.81 | 0.12 |
| 15.8 | **Expanded model** | 0.34 | 1.01 | 0.75 | 0.06 |
|  | Split sample validation | 0.18 | 1.28 | 0.78 | 0.07 |

*Abbreviations: DNN: deep neural network; MAE: mean absolute error; RMSE: root mean sum of squared errors.*

**Figure S1** Bland-Altman plots of ePWV versus measured-PWV. A: basic ePWV ridge regression model. B: expanded ePWV ridge regression model. C: basic ePWV DNN model. D: expanded ePWV DNN model.


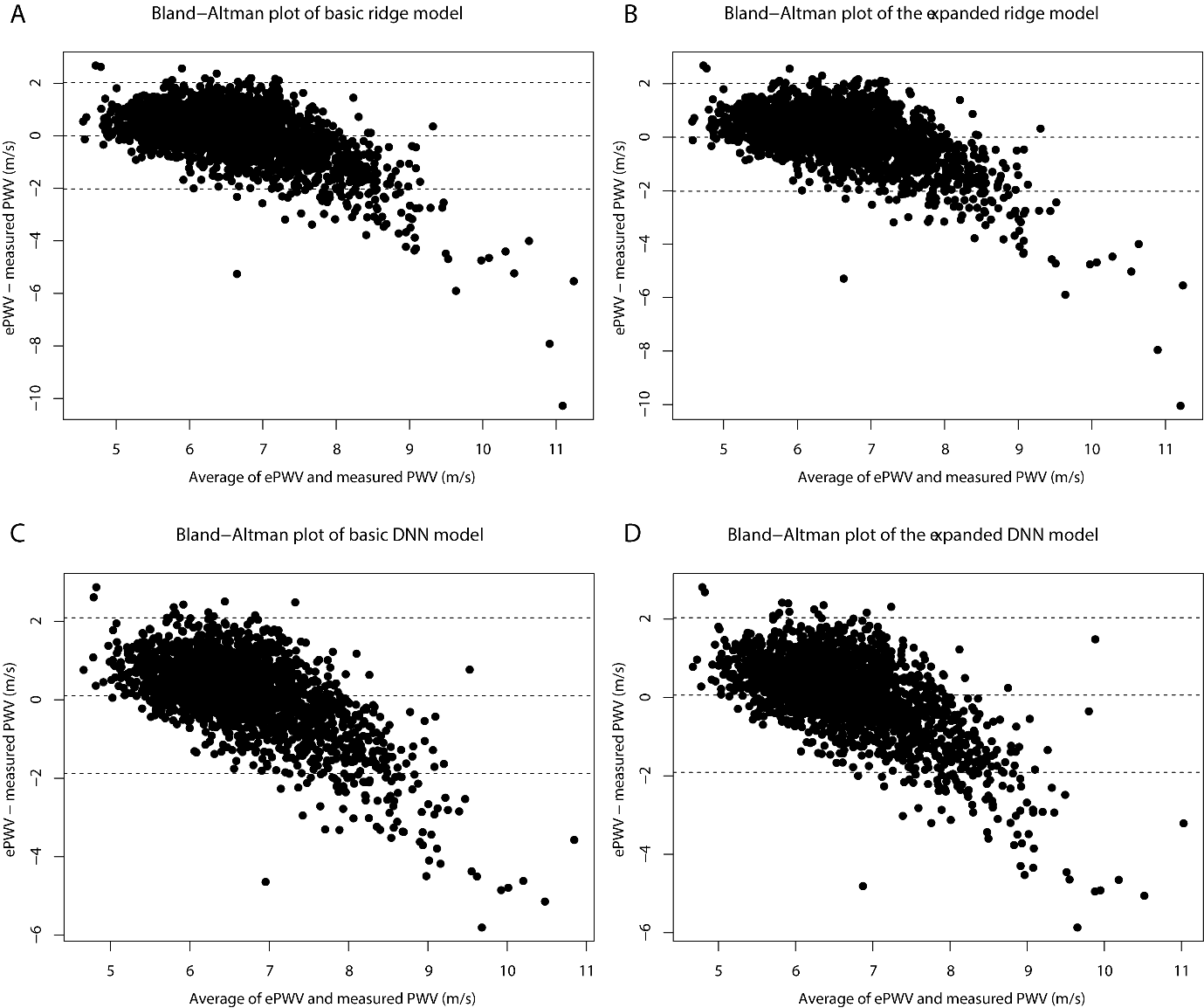

Supplement: Supplementary file 1 — Supplementary file1 (DOCX 265 kb) [file 10554_2021_2359_MOESM1_ESM.docx]
